# *FaAKR23* Modulates Ascorbic Acid and Anthocyanin Accumulation in Strawberry (*Fragaria × ananassa*) Fruits

**DOI:** 10.3390/antiox11091828

**Published:** 2022-09-16

**Authors:** Lingzhi Wei, Huabo Liu, Yang Ni, Jing Dong, Chuanfei Zhong, Rui Sun, Shuangtao Li, Rong Xiong, Guixia Wang, Jian Sun, Yuntao Zhang, Linlin Chang, Yongshun Gao

**Affiliations:** 1 Institute of Forestry and Pomology, Beijing Academy of Agriculture and Forestry Sciences, Beijing 100093, China; 2 Key Laboratory of Biology and Genetic Improvement of Horticultural Crops (North China), Ministry of Agriculture and Rural Affairs, Beijing 100093, China; 3 Beijing Engineering Research Center for Strawberry, Beijing 100093, China; 4 Inspection and Testing Laboratory of Fruits and Nursery Stocks (Beijing), Ministry of Agriculture and Rural Affairs, Beijing 100093, China

**Keywords:** strawberry, antioxidants, ascorbic acid, anthocyanin, AKR

## Abstract

Strawberry (*Fragaria × ananassa*) fruits are rich in ascorbic acid (AsA) and anthocyanin, which are essential antioxidants for human health. However, the underlying regulatory mechanism of these antioxidant accumulation, especially AsA accumulation in strawberry fruits, remains largely unknown. In this study, we identified *FaAKR23* was a regulator of AsA and anthocyanin accumulation. We transiently expressed *FaAKR23* in strawberry fruits and conducted metabolic and molecular analyses to explore the role of *FaAKR23* in AsA and anthocyanin accumulation. Transient silencing of *FaAKR23* (*FaAKR23*-RNAi) in strawberry fruits significantly decreased the AsA and anthocyanin contents compared with control (empty vector-RNAi, EV-RNAi). Correspondingly, expression of some structural genes and regulatory factors involved in these two antioxidants’ accumulation was dramatically repressed. In addition, transcriptome analysis of EV-RNAi and *FaAKR23*-RNAi fruits suggested that *FaAKR23* was also involved in starch and sucrose metabolism as well as plant–pathogen interaction. Overall, these results not only provide the coordinated regulatory function of *FaAKR23* on AsA and anthocyanin accumulation but also offer a promising candidate gene for strawberry breeding with high antioxidants.

## 1. Introduction

Strawberry (*Fragaria × ananassa*), a worldwide fruit, has excellent commercial value and health benefits [1]. Vitamin C (ascorbic acid, AsA) and anthocyanin are crucial antioxidants that not only play important roles in strawberry fruit quality formation but also contribute to human health and disease prevention [2]. However, the understanding of the regulation of AsA and anthocyanin accumulation, especially the AsA accumulation mechanism, remains largely unknown. Accordingly, screening and identifying the candidate genes involved in AsA and anthocyanin in strawberry fruits will provide new insights into the two antioxidants’ accumulation mechanisms and are of particular importance for antioxidant-rich strawberry breeding.

AsA is an important water-soluble antioxidant that is indispensable for eliminating reactive oxygen species (ROS), thereby keeping cell redox levels stable and maintaining normal activities in plants. The d-mannose/l-galactose (d-Man/l-Gal) pathway is the major pathway for AsA biosynthesis in higher plants [3,4], which consists of eight reaction steps catalyzed by phosphomannose isomerase (PMI), phosphomannose mutase (PMM), GDP-d-mannose pyrophosphorylase (GMP), GDP-d-mannose-3′,5′-epimerase (GME), GDP-l-galactose phosphorylase (GGP), l-galactose-1-phosphate phosphatase (GPP), l-galactose dehydrogenase (l-GalDH), and l-galactono-1,4-lactone dehydrogenase (l-GalLDH) [5]. In addition, the D-galacturonic acid (d-GalUA) pathway is another validated AsA biosynthetic pathway in plants; it was first identified in strawberry plants [5,6]. In 2003, researchers isolated the gene encoding d-GalUA reductase (*FaGalUR*) that catalyzes a key step in the d-GalUA pathway [6]. Overexpression of *FaGalUR* in *Arabidopsis*, potato, and tomato led to a 2~3-fold increase in AsA content in the transgenic plant leaves and fruits compared with control [6,7,8]. These results indicate that *FaGalUR* plays a significant role in AsA biosynthesis and can be used as a target to improve AsA content in strawberry fruits by genome editing [9,10].

Anthocyanin is the major pigment contributing to strawberry fruit coloring, which influences the commodity and nutritive value of strawberries. Moreover, anthocyanin is important antioxidant in plants, and it plays a key role in exogenous stress tolerance [11]. The biosynthesis of anthocyanin is carried out by a series of enzymes, including phenylalanine ammonia-lyase (PAL), cinnamate 4-hydroxylase (C4H), 4-coumarate coenzyme A ligase (4CL), chalcone synthase (CHS), chalcone isomerase (CHI), flavonoid 3-hydroxylase (F3H), dihydroflavonol-4-Reductase (DFR), anthocyanidin synthase (ANS), and UDP-glycose flavonoid 3-O-glycosyltransferase (UFGT); they cooperatively synthesize anthocyanin [12]. To date, a number of transcription factors have been verified to participate in the accumulation of anthocyanin in strawberries, such as *FaMYB10*, *FaGAMYB*, *FaMYB9*/*11*, *FabHLH3*, *FaTTG1*, *FaRAV1*, *FaMADS1a*, *FaMADS9*, *FaRAP,* and *FaRIF* [13,14,15,16,17,18,19,20,21]. Besides these transcription factors, some important regulatory proteins regulate the level of anthocyanin, namely, protein phosphatase ABI1, protein kinase FaSnRK2.6, FaMRLK47, and FvMAPK3 [22,23,24,25].

Aldo-keto reductases (AKRs) are a superfamily of NADH-dependent oxidoreductases, including aldehyde reductase, aldose reductase and xylose reductase [26]. AKRs are found in animals, plants, and bacteria and regulate cell oxidation reduction (redox) under multiple stress conditions. In mice, *AKR1A* performs the function of glucuronolactone reductase; knockout *AKR1A* induces the lack of AsA in mice [27], which indicates that *AKR1A* might participate in AsA biosynthesis in mice. In plants, AKRs plays an important role in the accumulation of antioxidants and stress tolerance [28,29]; for instance, AKR family member *FaGalUR* is an important enzyme in the d-GalUA pathway for AsA synthesis [6]. Oberschall et al. cloned a NADPH-dependent AKR family gene, *MsALR,* in alfalfa; the heterologous expression of *MsALR* in tobacco significantly improved its tolerance to herbicide and redox damage caused by heavy metals [29]. In rice and tobacco, the overexpression of *PsAK1R* or *OsAKR1* prominently increased herbicide resistance [30], and the transgenic tobacco contained higher glutathione, reducing the H_2_O_2_ level, elevating the chlorophyll content, and, finally, enhancing salt tolerance [31]. In peach (*Prunus persica*), expression of the aldo-keto reductase gene *PpAKR1* was induced by ABA, oxidative stress, low temperature, and salt stress; furthermore, *PpAKR1* enhanced salt resistance [32].

According to our previous work, *FaAKR23* was identified as the most dramatically increased *FaAKR* gene during fruit development and ripening in cultivated strawberries [33]. However, little is known about how it regulates fruit ripening and quality formation, especially antioxidant accumulation. In this study, we first clarify that *FaAKR23* is a regulator of AsA and anthocyanin accumulation in strawberry fruits. The results not only provide the coordinated regulatory function of *FaAKR23* on AsA and anthocyanin accumulation but also offer a promising candidate gene for strawberry breeding with high antioxidants.

## 2. Materials and Methods

### 2.1. Plant Materials and Growth Conditions

Octoploid strawberries (*F. × ananassa* cv. ‘Sweet Charlie’) were grown in a greenhouse under natural sunlight conditions at the Institute of Forestry and Pomology, Beijing Academy of Agriculture and Forestry Sciences, Beijing, China. Fruit ripening stages were classified based on days post-anthesis (DPAs), including small green (SG), big green (BG), white (W), turning (T), and red (R) [22]. The collected fruit samples of all the five stages were immediately frozen in liquid nitrogen and stored at −80 ℃ for subsequent analysis.

### 2.2. Phylogenetic Analysis and Sequence Alignment

Based on our previous study [33], the FaAKR23 and FaAKR24 protein sequences were downloaded from the Genome Database for Rosaceae (GDR, https://www.rosaceae.org, accessed on 1 January 2022), and the reported AKR proteins in *Arabidopsis thaliana*, *Oryza sativa* L., and *Prunus persica* were acquired from NCBI. The phylogenetic tree was constructed via the neighbor-joining method in MEGA version 7.0 software with 1000 bootstrap replicates. The protein sequence alignment was performed using Clustal X version 2.1 with default parameters [34] The sequences used to construct the phylogenetic trees and sequence alignment are listed in Table S1.

### 2.3. Protein Subcellular Localization

The full-length coding sequence (CDS) of *FaAKR23* without a stop codon was amplified by PCR, cloned into the pCAMBIA-super1300 vector, and then transformed into *Agrobacterium tumefaciens* strain GV3101. Agrobacteria cultures were resuspended in infiltration buffer to a final OD_600_ = 0.6 and then were infiltrated into epidermal cells of 4- to 6-week-old tobacco leaves. The infiltrated tobacco leaves were incubated at 23 ℃ under an 8 h/16 h light/dark cycle for 48–72 h; after that, the leaves were subjected to green fluorescent protein fluorescence observation using a confocal laser scanning microscope (Nikon A1). The primers used for cloning are listed in Table S2.

### 2.4. Transient Expression of FaAKR23 in Strawberry Fruits

The overexpression vector pCAMBIA-super1300 and RNAi vector pFGC5941 were used to overexpress and silence *FaAKR23*. The full-length *FaAKR23* CDS was PCR amplified from the strawberry genome and recombined into the pCAMBIA-super1300 vector. To construct the *FaAKR23*-RNAi vector, two fragments of the *FaAKR23* CDS were inserted into vector pFGC5941 in the opposite orientation on either side of the chalcone synthase A intron. *FaAKR23*-OE, *FaAKR23*-RNAi, and empty vectors (EV-OE and EV-RNAi) were introduced into the *Agrobacterium tumefaciens* strain GV3101, and the agrobacterium cells were cultured at 28 °C in LB medium. The agrobacterium cultures were resuspended in infiltration buffer (10 mM MES (pH 5.6), 10 mM MgCl_2_, and 100 µM acetosyringone) and adjusted to OD_600_ = 0.8. The agrobacterium suspension was injected in the octoploid fruits at the BG stage, with consistent growth status when they became hygrophanous. After 7 days, these injected fruits were collected and frozen in liquid nitrogen immediately and then stored at −80 °C for further analysis. For each individual experiment, three biological repeats were performed; each repeat consisted of 10 fruit individuals. The primers used in vector construction are provided in Table S2.

### 2.5. Anthocyanin and AsA Analysis

Strawberry fruits transient expression of *FaAKR23* or empty vector was ground to powder under liquid nitrogen. Approximately 1 g powder was mixed with 5 mL methanol-0.05% HCl and then extracted at 4 °C under dark for 12 h. The supernatant was collected by centrifugation for further analysis. Anthocyanin content was determined by the pH-differential method using buffer solutions of sodium acetate (0.4 M, pH 1.0) and potassium chloride (0.025 M, pH 4.5), and the absorbance was read at 530 and 700 nm, respectively [19].

The total AsA content of strawberry fruits was determined by high-performance liquid chromatography (HPLC) (Waters 2695). Briefly, 5 g frozen samples in powder were suspended in 2% (*w/v*) metaphosphoric acid and 2 mM EDTA, chilled on ice for 20 min, and centrifuged at 13,000 rpm for 20 min at 4 °C. The 15 µL filtered supernatant was injected into the HPLC system. The mobile phase consisted of 0.1 M sodium acetate/acetic acid buffer and 5% methanol (pH 5.8), and AsA was detected using a C18 5 µm 25 × 0.46 column. Total AsA was calculated by comparison with the standard curve using the peak areas at 254 nm [35].

### 2.6. RNA Extraction and qRT-PCR Analysis

Total RNA was extracted from strawberry fruits using an E.Z.N.A.^®^ Total RNA Kit (Omega, Norcross, GA, USA) according to the manufacturer’s instructions. The first-strand cDNA was synthesized from 1 μg total RNA with EasyScript^®^ First-Strand cDNA Synthesis SuperMix (Transgene, Beijing, China). The qRT-PCR was performed on a CFX96TM Real-Time System (Bio-Rad, USA) using TB GreenTM Premix Ex TaqTM II (Takara, Japan). *FaCHP1* was used as the internal reference to standardize the transcript level [36]. Relative expression was calculated using the 2^−ΔΔCt^ method. Three biological replicates were repeated. The primers are listed in Table S3.

### 2.7. Transcriptome Deep Sequencing (RNA-Seq) Analysis

Total RNA was extracted from the strawberry fruits of EV-RNAi and *FaAKR23*-RNAi using the E.Z.N.A.^®^ Total RNA Kit (Omega, Norcross, GA, USA). RNA concentration and purity were measured using Nano Drop 2000 (Thermo Fisher Scientific, Wilmington, DE). RNA integrity was assessed using the RNA Nano 6000 Assay Kit of the Agilent Bioanalyzer 2100 system (Agilent Technologies, Palo Alto, CA, USA). Sequencing was performed on an Illumina genome analyzer at the Biomarker Technologies Corporation. Genes with an adjusted FDR of <0.01, as determined by DESeq2, were considered differentially expressed [37]. Three independent biological replicates were analyzed in this experiment. The raw data of the RNA-seq has been submitted to the NCBI database (accession number: PRJNA869890; http://www.ncbi.nlm.nih.gov/sra, accessed on 15 August 2022).

### 2.8. Statistical Analysis

Statistical analysis was performed with GraphPad Prism (version 8.0); statistically significant differences between samples were calculated using two-tailed Student’s *t*-tests (* *p* < 0.05, ** *p* < 0.01).

## 3. Results

### 3.1. Identification and Characterization of FaAKR23 in Cultivated Strawberry

**Figure 1:**
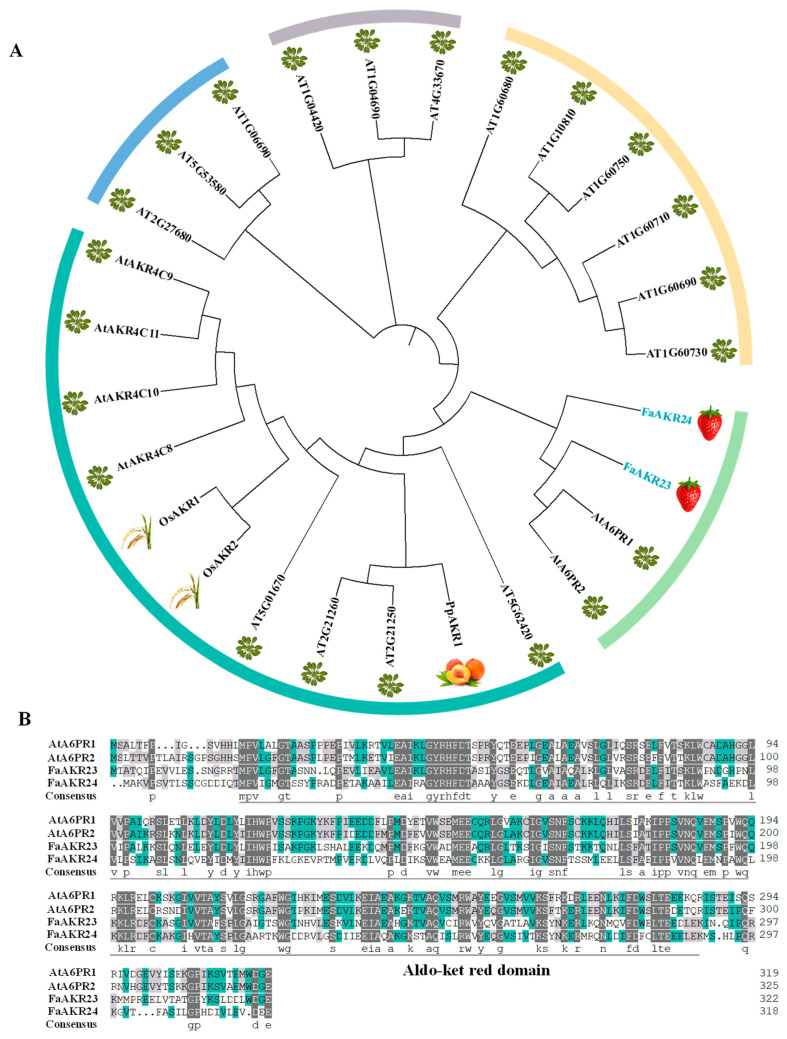
Phylogenetic analysis and multiple sequence alignment of AKR proteins. (**A**) Phylogenetic analysis of AKRs. At, *Arabidopsis thaliana*; Fa, *Fragaria × ananassa*; Os, *Oryza sativa*.L; Pp, *Prunus persica*. The full-length protein sequences are provided in Table S1; (**B**) multiple sequence alignment was performed using the protein sequences of FaAKR23, FaAKR24, AtA6PR1, and AtA6PR2; the Aldo-ket red domain is labeled by a gray line.

In our previous work, we identified that the transcript level of *FaAKR23* was the highest in receptacles and that its expression levels increased by 142-fold from the green fruit stage to the red fruit stage, followed by the *FaAKR24*, which is the homologous gene of *FaGalUR* [33]. In this study, we further investigate the function of *FaAKR23*. To analyze the evolutionary relationship among these reported AKR members in *Arabidopsis thaliana*, *Oryza sativa* L., *Prunus persica*, and *Fragaria* × *ananassa* [32,33,38,39,40], a maximum likelihood (ML) tree was constructed using MEGA 7. The results showed that all AKRs were divided into five subgroups, and FaAKR23, FaAKR24, AtA6PR1, and AtA6PR2 were located in the same group, implying a partly similar function among them (Figure 1A). Furthermore, the protein sequence alignment of AtA6PR1, AtA6PR2, FaAKR23, and FaAKR24 suggest that these four proteins are harboring the conserved motif of the Aldo-ket red domain (Figure 1B); another study has indicated that AtA6PR1 and AtA6PR2 are cytosolically localized and respond differentially to cold and salt stresses [40].

**Figure 2:**
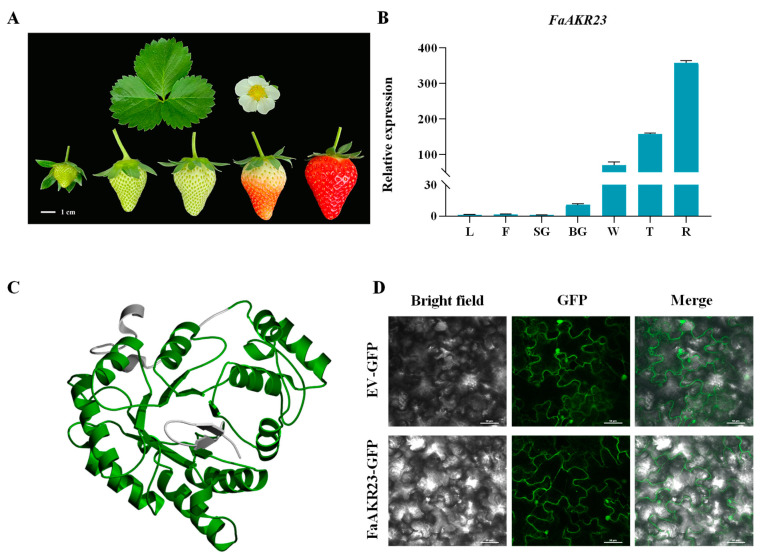
The phenotypes of different tissues in strawberry, expression patterns, protein structure, and subcellular localization of FaAKR23. (**A**) Phenotypes of strawberry leaf, flower, and fruits in different development stages. (**B**) The transcript levels of *FaAKR23* in different strawberry tissues. L, leaf, F, flower, SG, small green fruit stage, BG, big green fruit stage, W, white fruit stage, T, turning stage, R, fully red stage. (**C**) Protein structure of FaAKR23. (**D**) Subcellular localization of FaAKR23. Scale bars: 50 μm.

To investigate the potential function of *FaAKR23*, we firstly examined the *FaAKR23* transcription levels in different tissues of octoploid strawberry *F. × ananassa* cv. ‘Sweet Charlie’ (Figure 2A). As shown in Figure 2B, *FaAKR23* transcript levels were present at much lower levels in leaves and flowers than in fruits, and its expression increased extremely during fruit development until ripening; this implies that *FaAKR23* might be involved in strawberry fruit ripening and quality formation. In addition, the protein structure predicted by SWISS-MODEL showed that the FaAKR23 protein folds into a TIM barrel structure, which is highly conserved during evolution [41] (Figure 2C).

### 3.2. Subcellular Localization of FaAKR23

To examine the subcellular localization of the FaAKR23 protein, the full-length of the FaAKR23 protein was fused with the N-terminal of the green fluorescent protein (GFP) tag, driven by the CaMV 35S promoter. We transiently expressed the FaAKR23-GFP fusion protein in tobacco leaf cells. Visualization of the fused protein showed that FaAKR23 has a dual localization in both nucleus and cytoplasm (Figure 2D).

### 3.3. FaAKR23 Regulates Anthocyanin and AsA Accumulation in Strawberry Fruits

**Figure 3:**
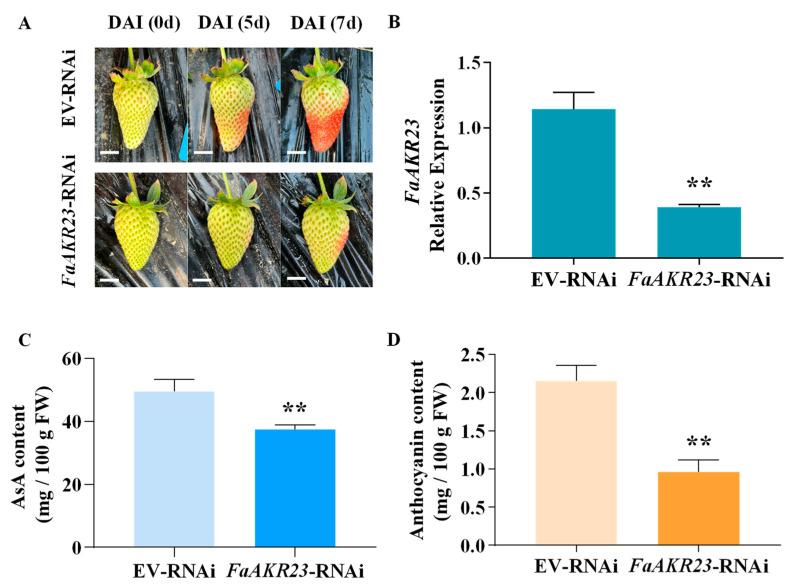
Transient silenced expression of *FaAKR23* in strawberry fruits. (**A**) Phenotypes of EV-RNAi and *FaAKR23*-RNAi fruits; EV, empty vector; scale Bars: 1 cm. (**B**) The expression levels of *FaAKR23* in EV-RNAi and *FaAKR23*-RNAi fruits. Expression values are means ± SEMs of three biological replicates and are normalized using *FaCHP1* as an internal control. (**C**) AsA content in EV-RNAi and *FaAKR23*-RNAi fruits. (**D**) Anthocyanin content in EV-RNAi and *FaAKR23*-RNAi fruits. Statistically significant differences from control were determined by Student’s *t*-test: ** *p* < 0.01. Values are means ± SEMs of three biological replicates.

To investigate the potential role of *FaAKR23* in strawberry fruit ripening and quality formation, we transiently expressed *FaAKR23* in octoploid strawberry fruits via agroinfiltration. The visual phenotype suggested that *FaAKR23* was involved in anthocyanin accumulation (Figure 3A). Using qRT-PCR analysis, we confirmed the lower relative *FaAKR23* mRNA level in *FaAKR23*-RNAi transiently transformed strawberry fruits (Figure 3B). Furthermore, the AKR family is widely reported to regulate cell redox levels in both animals and plants [28] because AsA and anthocyanin are important antioxidants in strawberry fruits. Hence, we further determined the AsA and anthocyanin contents in EV-RNAi and *FaAKR23*-RNAi transformed fruits, and it was found that the contents of both AsA and anthocyanin were obviously reduced in the *FaAKR23*-RNAi fruits compared to control; the AsA level decreased by approximately 30%, and the anthocyanin content was reduced by nearly 50% (Figure 3C,D).

### 3.4. Expression Patterns of AsA and Anthocyanin Metabolism-Related Genes in EV-RNAi and FaAKR23-RNAi Fruits

**Figure 4:**
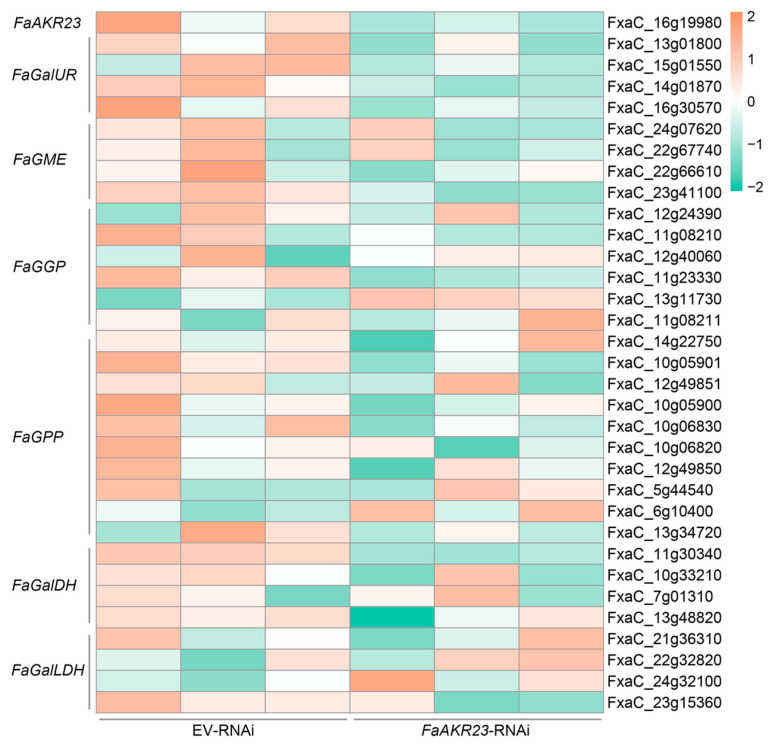
Expression profiles of AsA metabolism-related genes differently expressed between EV-RNAi and *FaAKR23*-RNAi fruits.

**Figure 5:**
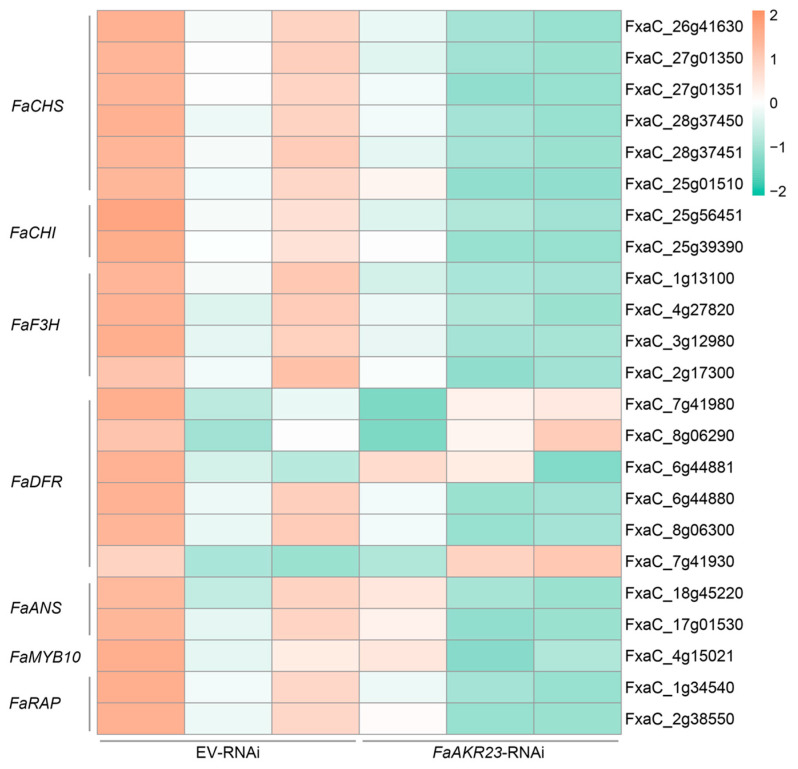
Expression profiles of anthocyanin biosynthesis genes differently expressed between EV-RNAi and *FaAKR23*-RNAi fruits.

**Figure 6:**
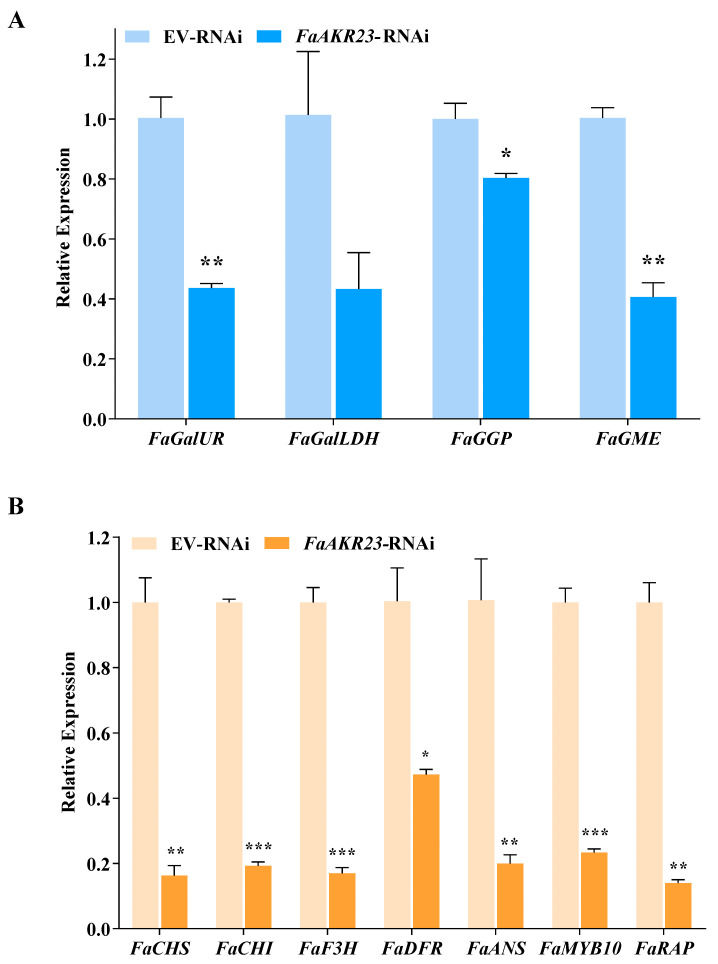
Validation of relative expression levels of AsA metabolism-related and anthocyanin biosynthetic genes in EV-RNAi and *FaAKR23*-RNAi strawberry fruits by qRT-PCR. *FaCHP1* was used as an internal reference. Values are means ± SEMs of three biological replicates. Statistical significance was determined by Student’s *t*-test: * *p* < 0.05; ** *p* < 0.01; *** *p* < 0.001.

To decipher the molecular mechanisms underlying *FaAKR23*-mediated AsA and anthocyanin accumulation in strawberry fruits, we performed transcriptome deep sequencing (RNA-seq) using EV-RNAi and *FaAKR23*-RNAi fruits. FDR < 0.01 and fold change ≥ 2 were set as the threshold for significantly differential expression (Figure 4 and Figure 5). We also validated the expression of several genes of interest by qRT-PCR (Figure 6). These results showed that the AsA biosynthetic genes, such as *FaGalUR*, *FaGME*, and *FaGGP* (Figure 4), and anthocyanin-synthesis-related genes, such as *FaCHS*, *FaCHI*, *FaF3H*, *FaDFR*, and *FaANS*, as well as *FaMYB10* and *FaRAP*, were significantly downregulated (Figure 5).

### 3.5. FaAKR23 Involved in Plant–Pathogen Interaction and Starch and Sucrose Metabolism

Kyoto Encyclopedia of Genes and Genomes (KEGG) classification of differentially expressed genes (DEGs) in EV-RNAi and *FaAKR23*-RNAi fruits showed that many DEGs were involved in plant–pathogen interaction, starch and sucrose metabolism, and the MAPK signaling pathway, which are closely associated with fruit development and quality formation (Figure S2). Furthermore, the expression of 23 *FaWRKY* transcription factor genes were significantly upregulated in FaAKR23-RNAi transiently transformed fruits, and 3 *FaWRKY* members were downregulated (Figure S3). These results hint that *FaAKR23* might play multiple functions during strawberry fruit development and ripening.

## 4. Discussion

The AKR superfamily is a large enzyme group of oxidoreductases that mainly use NADP(H) as a cofactor; this group is widely distributed in animals, plants, yeast, and bacteria and regulates cell oxidation reduction under multiple stress conditions. Although AKRs play numerous roles in the metabolism of steroids, sugars, and other carbonyls, many AKR superfamily members remain uncharacterized [39].

In octoploid strawberry, a total of 80 *FaAKR* genes were identified from the genome and divided into 20 subgroups [33]. Among the members, *FaGalUR* (*FaAKR24*) has been characterized and proven to play an important role in AsA biosynthesis [6]. Overexpression of *FaGalUR* in *Arabidopsis*, potato, and tomato leads to a 2~3-fold increase in AsA content in the transgenic plant leaves and fruits compared with the wild-type [6,7,8]. However, whether other AKR genes in strawberry also contribute to AsA accumulation or fruit ripening is not known. Recently, we performed a genome-wide analysis of the AKR superfamily in cultivated strawberry and found that *FaAKR23* was the most extremely upregulated *FaAKR* gene during fruit development and ripening [33] (Figure 2B). Moreover, *FaAKR23* and *FaGalUR* (*FaAKR24*) were in a close phylogenetic relationship, and *AtA6PR1* and *AtA6PR2* were also located in the same cluster (Figure 1A). Previously, *AtA6PR1* and *AtA6PR2* were proposed to be related to the aldose 6-phosphate reductase pathway, which is involved in sorbitol synthesis. Sorbitol is the main form of photosynthetically fixed carbon, translocated via the phloem from source to sink organs in plants [40]; consequently, we speculated that *FaAKR23* might be involved in strawberry fruit ripening and quality formation.

To confirm this deduction, we manipulated *FaAKR23* by transient expression in strawberry fruits and conducted metabolic and molecular analyses. The results demonstrated that *FaAKR23* is indeed involved in AsA and anthocyanin accumulation through the regulation of the transcription level of downstream structural genes as well as some regulator factors of these two metabolic pathways. AsA content obviously decreased in the *FaAKR23*-RNAi fruits (Figure 3C). The RNA-seq and qPCR analyses showed that the expression of multiple AsA biosynthetic genes were significantly suppressed, especially *FaGalUR*, *FaGGP,* and *FaGME* (Figure 4 and Figure 6A). Interestingly, we found that the FaAKR23 protein was located in both cytoplasm and nuclear, which suggests that *FaAKR23* might work as a transcription factor (Figure 2D). To date, a number of TFs have been reported to be involved in AsA biosynthesis. These include the AP2 ethylene response factors *ERF98* [42]; *ABI4* [43], the basic helix–loop helix *bHLH59* [44], the HD-Zip family TF *SlHZ24* [45], and SlNFYA10 [46] bind to *VTC1*, *GGP,* and *GME* to promote or reduce AsA synthesis. More recently, in kiwifruit, ethylene response factor *AcERF91* has been found to increase the expression of *AcGGP3* [47], and transcription factors *AceMYBS1* and *AceGBF3* influence AsA biosynthesis by activating the transcription of *AceGGP3* [48]. Consistent with this knowledge, it is necessary that the specific regulatory mechanism of *FaAKR23* in AsA accumulation be further clarified.

In addition, anthocyanin was also dramatically decreased in *FaAKR23*-RNAi fruits (Figure 3D), along with lower expression of anthocyanin biosynthetic genes and regulatory factors (Figure 5 and Figure 6B). In *FaAKR23*-RNAi transcriptome data and qRT-PCR analysis, the transcript levels of anthocyanin biosynthetic genes *FaCHS*, *FaCHI*, *FaF3H*, *FaDFR*, and *FaANS* were significantly repressed. Moreover, the expression of *FaMYB10* was decreased in *FaAKR23*-RNAi fruits; it is a powerful and conserved regulator of anthocyanin biosynthesis in diploid and octoploid strawberry fruits [12,13,14,21] and also regulates the expression of *CHS1*, *CHI*, *DFR*, *F3H*, and *DFR* individuals by forming an MYB–bHLH–WD complex, thereby regulating anthocyanin accumulation [13]. To date, a number of regulatory factors involved in modulating anthocyanin accumulation in strawberry fruits has been characterized, including *FaRAV1*, *FaRAP*, *FaSnRK2.6*, *FaMADS9*, *FaRIF*, *FaMYB9/MYB11*, and *FaGAMYB* [13,14,15,16,17,18,19,20,21]. Besides these transcription factors, some important regulatory proteins downregulate the level of anthocyanin, such as protein phosphatase ABI1, protein kinase FaSnRK2.6, FaMRLK47, and FvMAPK3 [22,23,24,25]. Taken together, the key question that needs to be addressed is whether the interplay between *FaAKR23* and *FaMYB10* results in anthocyanin accumulation or whether FaAKR23 regulates anthocyanin by directly binding to the promoter of *FaMYB10.*

Nevertheless, when *FaAKR23* is overexpressed in strawberry fruits, *FaAKR23*-OE fruits do not show higher content of either AsA or anthocyanin compared to EV-OE fruits (Figure S1). We speculated that, for one thing, there might be a precisely controlled mechanism of AsA and anthocyanin accumulation that protects the plants from overloading the antioxidants, as, even though *GGP* or *GalUR* genes are overexpressed in plants, the AsA level did not increase as remarkably as that of gene expression; in some cases, AsA was only moderately promoted [49,50]. Moreover, the nuclear-located FaAKR23 possibly needs other cooperative factors, such as transcription factor *FaMYB10*, to improve the biosynthesis of the two antioxidants.

Furthermore, RNA-seq data revealed that many DEGs were involved in plant–pathogen interaction, starch and sucrose metabolism, and the MAPK signaling pathway. We discovered here that the expression of 23 *FaWRKY* transcription factors was significantly upregulated and that 3 *FaWRKY* members were downregulated in *FaAKR23*-RNAi transiently transformed fruits (Figure S3). *WRKY* genes compose one of the largest families of transcription factors in land plants and are characterized by the highly conserved WRKY domain; WRKY proteins have been reported to play key roles in regulating growth, development, and biotic and abiotic stresses [51,52,53,54]. In strawberry, *FaWRKY1* has been characterized and involved in mediating defense responses to the fungus *Colletotrichum acutatum* [55]; moreover, FvWRKY48 binds to the pectate lyase *FvPLA* promoter to control fruit softening [56]. These results hint that *FaAKR23* may play multiple roles in strawberry in addition to AsA and anthocyanin accumulation in fruits.

To sum up, the specific regulatory mechanism of *FaAKR23* that modulates downstream structural genes and regulatory factors should be addressed, and the interacting proteins that assist *FaAKR23* in regulating AsA and anthocyanin content need to be further identified. In addition, whether plant hormone or abiotic stress acts as upstream signaling to regulate the expression of *FaAKR23* and then is involved in AsA and anthocyanin accumulation needs further research evidence to be clarified. Overall, our study provides new clues about the regulatory mechanisms of AsA and anthocyanin accumulation and offers a promising candidate gene for strawberry breeding with high antioxidants.

## 5. Conclusions

AsA and anthocyanin are crucial antioxidants that not only maintain the normal growth and development of plants but also contribute to human health and disease prevention. From this study, we demonstrate, for the first time, that *FaAKR23* is a regulator of AsA and anthocyanin accumulation in strawberry fruits by metabolic and molecular analyses. From the transcriptome data and qRT-PCR analysis, the transcript levels of the AsA and anthocyanin metabolism-related genes were significantly regulated in the *FaAKR23*-RNAi fruits compared with EV-RNAi fruits. Moreover, *FaAKR23* might be involved in the starch and sucrose metabolism and plant–pathogen interaction. Finally, this study provides new genetic clues for elucidating the regulation mechanism of AsA and anthocyanin accumulation. However, further research needs to be conducted on the specific molecular mechanism of *FaAKR23*-mediated AsA and anthocyanin accumulation in strawberry fruits.

## Data Availability

The data presented in this study are available in the article and Supplementary Materials.

## Supplementary Materials

The following supporting information can be downloaded at: https://www.mdpi.com/article/10.3390/antiox11091828/s1, Figure S1: Transient overexpression of *FaAKR23* in strawberry fruits. Figure S2: Kyoto Encyclopedia of Genes and Genomes (KEGG) classification of DEGs in EV-RNAi and *FaAKR23*-RNAi fruits; Figure S3: Transcriptome expression heatmap of *FaWRKYs* in EV-RNAi and *FaAKR23*-RNAi fruits; Table S1: GeneBank accession numbers and protein sequences used in phylogenetic tree construction; Table S2: Primers used in vector construction; Table S3: Primers used in qRT-PCR analysis.
