# Supplementary material for: FaAKR23 Modulates Ascorbic Acid and Anthocyanin Accumulation in Strawberry (Fragaria × ananassa) Fruits
Source: Antioxidants (Basel). 2022 Sep 16;11(9):1828. doi: 10.3390/antiox11091828 (PMC9495909; doi:10.3390/antiox11091828)
Supplement: Supplementary file 1 [file antioxidants-11-01828-s001.zip › Supplemental_Figures S1-S3.pdf]

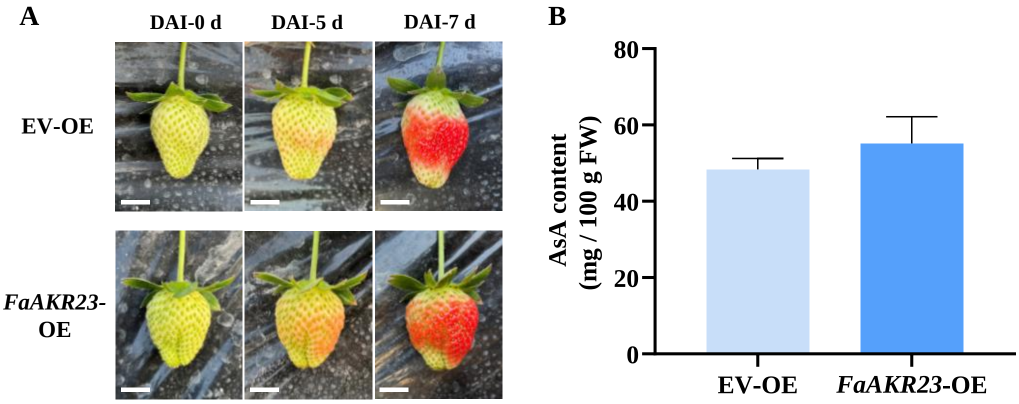

Supplemental Figure S1. Transient overexpression of *FaAKR23* in strawberry fruits. **(A)** Phenotypes of EV-OE and *FaAKR23*-OE fruits, EV, empty vector, Bars: 1 cm. **(B)** AsA content in EV-OE and *FaAKR23*-OE fruits.

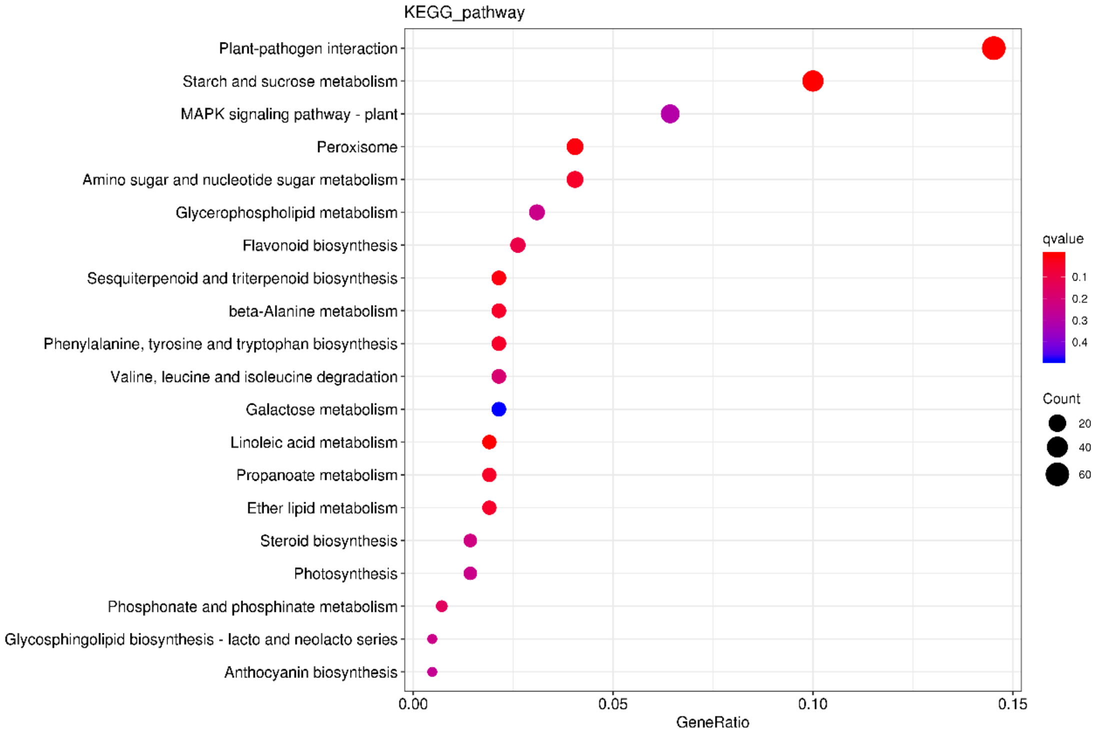

Supplemental Figure S2. Kyoto Encyclopedia of Genes and Genomes (KEGG) classification of DEGs in EV-RNAi and *FaAKR23*-RNAi fruits.

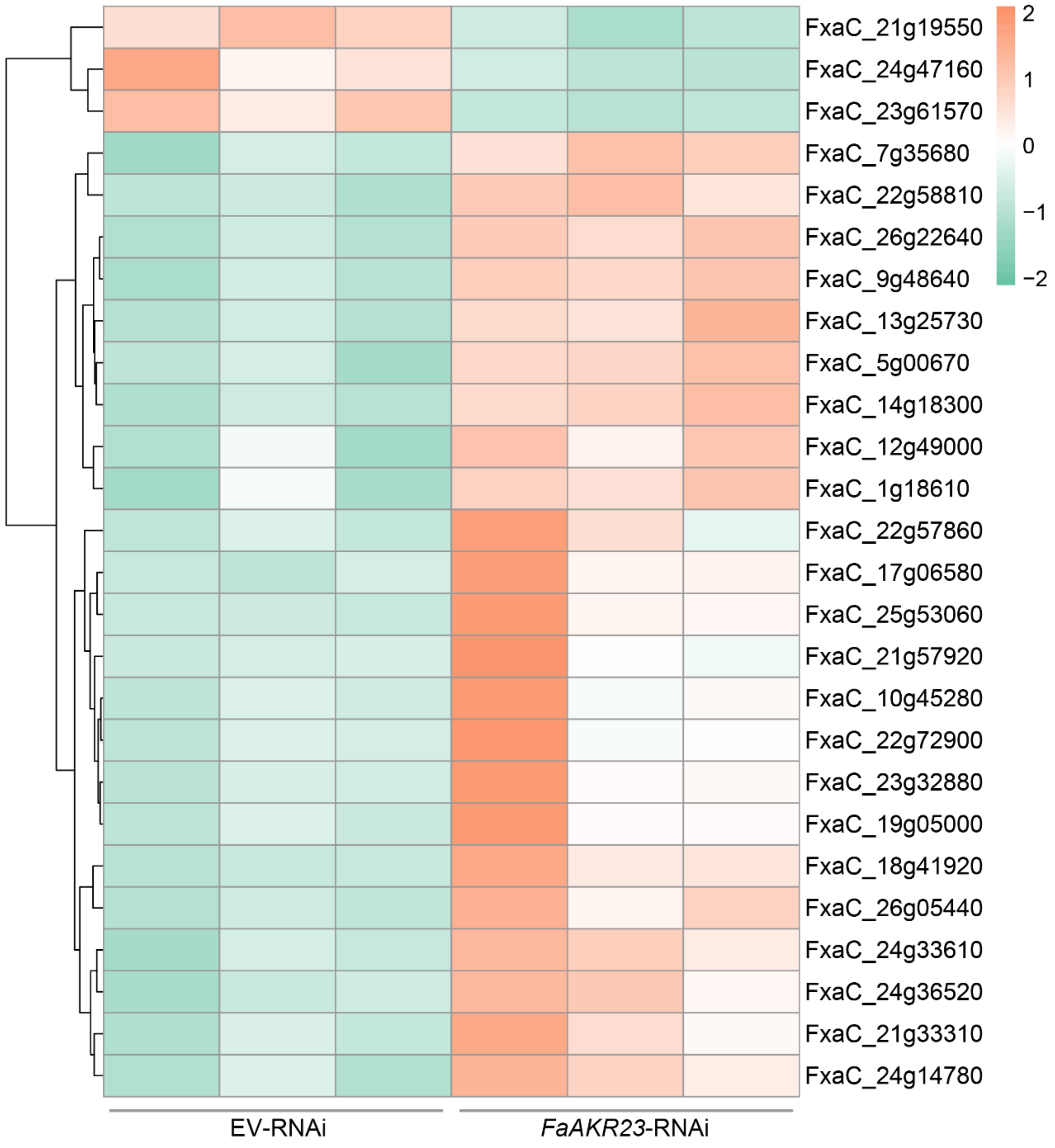

Supplemental Figure S3. Transcriptome expression heatmap of *FaWRKYs* in EV-RNAi and *FaAKR23*-RNAi fruits
